# *De novo* transcriptome assembly for the spiny mouse (*Acomys cahirinus*)

**DOI:** 10.1038/s41598-017-09334-7

**Published:** 2017-08-21

**Authors:** Jared Mamrot, Roxane Legaie, Stacey J. Ellery, Trevor Wilson, Torsten Seemann, David R. Powell, David K. Gardner, David W. Walker, Peter Temple-Smith, Anthony T. Papenfuss, Hayley Dickinson

**Affiliations:** 1 The Ritchie Centre, Hudson Institute of Medical Research, Melbourne, Australia; 2 Department of Obstetrics and Gynaecology, Monash University, Melbourne, Australia; 3 MHTP node - Monash Bioinformatics Platform, Monash University, Melbourne, Australia; 4 MHTP Medical Genomics Facility, Melbourne, Australia; 5 Melbourne Bioinformatics, The University of Melbourne, Melbourne, Australia; 6 Monash Bioinformatics Platform, Monash University, Melbourne, Australia; 7 School of BioSciences, University of Melbourne, Melbourne, Australia; 8 Education Program in Reproduction and Development, Monash University, Melbourne, Australia; 9 Bioinformatics Division, Walter and Eliza Hall Institute, Parkville, Australia; 10 Computational Cancer Biology Program, Peter MacCallum Cancer Centre, Melbourne, Australia; 11 Department of Medical Biology, University of Melbourne, Melbourne, Australia; 12 Sir Peter MacCallum Department of Oncology, University of Melbourne, Melbourne, Australia; 13 RMIT University, Bundoora Campus, Bundoora, Australia

## Abstract

Spiny mice of the genus *Acomys* display several unique physiological traits, including menstruation and scar-free wound healing; characteristics that are exceedingly rare in mammals, and of considerable interest to the scientific community. These unique attributes, and the potential for spiny mice to accurately model human diseases, are driving increased use of this genus in biomedical research, however little genetic information is accessible for this species. This project aimed to generate a draft transcriptome for the Common spiny mouse (*Acomys cahirinus*). Illumina sequencing of RNA from 15 organ types (male and female) produced 451 million, 150 bp paired-end reads (92.4Gbp). An extensive survey of *de novo* transcriptome assembly approaches using Trinity, SOAPdenovo-Trans, and Oases at multiple kmer lengths was conducted, producing 50 single-kmer assemblies from this dataset. Non-redundant transcripts from all assemblies were merged into a meta-assembly using the EvidentialGene *tr2aacds* pipeline, producing the largest gene catalogue to date for *Acomys cahirinus*. This study provides the first detailed characterization of the spiny mouse transcriptome. It validates use of the EvidentialGene *tr2aacds* pipeline in mammals to augment conventional *de novo* assembly approaches, and provides a valuable scientific resource for further investigation into the unique physiological characteristics inherent in the genus *Acomys*.

## Introduction

The Common or Cairo spiny mouse (*Acomys cahirinus*) is a small rodent species endemic to the semi-arid deserts of Africa and the Middle East^1^. Used in research to model human disease, spiny mice exhibit physiological characteristics not typically found in rodents: they exhibit a precocial pattern of development^2, 3^, atypical synthesis of hormones such as cortisol and dehydroepiandosterone^4–6^, and a menstrual cycle^7^. These traits are common to humans and other higher order primates, but rare in other mammals. For example, menstruation has been identified in only six non-primate species (from >5,000 extant mammals), none of which are rodents^8^. The discovery of human-like physiological characteristics in a rodent is highly valuable for those in the scientific community looking to model human conditions, however fundamental aspects of their biology remain unexplored; for instance, there is little genetic information accessible for this species.

Publically available genetic information for the spiny mouse consists of the mitochondrial genome^9^, and two RNA sequencing (RNA-Seq) datasets: PRJNA184055^10^, and PRJNA292021^11^. These next-generation sequencing (NGS) datasets were created with specific aims: to establish incipient sympatric speciation as a mode of natural selection in mammals inhabiting divergent microclimates^9^, to examine the molecular basis for natural variation in mammalian lifespan^10^, and to characterize and investigate another characteristic unique to *Acomys*: scar-free wound healing and skin regeneration^11^. *De novo* assembly of NGS reads was conducted for each specific organ/tissue sequenced in these projects in order to investigate differential gene expression, however the accuracy and completeness of resulting assemblies was not explicitly described. Accurate identification of differentially expressed genes is dependent on accurate read mapping^12^, and an accurate reference assembly requires transcripts from multiple organ types.

Here, we describe a survey of *de novo* transcriptome assembly methods, utilizing both single-kmer and multi-kmer approaches, with the aim to generate a comprehensive *de novo* transcriptome assembly for the Common spiny mouse (*Acomys cahirinus*).

## Results

### Sample preparation and sequencing

Tissues were collected from male (n = 1), non-pregnant female (n = 1) and placenta from 2 pregnant female (1 male fetus, 1 female fetus) adult spiny mice in accordance with the Australian Code of Practice for the Care and Use of Animals for Scientific Purposes with approval from the Monash Medical Centre Animal Ethics Committee. Total RNA was extracted from skin, lung, liver, small intestine, kidney, adrenal gland, brain, thymus, spleen, diaphragm, heart, skeletal muscle (male only), testis (male only), ovary (female only), and placenta. All samples returned RNA integrity numbers (RIN scores) >7.0 and were pooled for sequencing. Samples were not multiplexed at the time of sequencing due to cost. Each tissues RNA sample is stored individually and able to be resequenced. The Illumina HiSeq. 1500 was used to produce 150 bp paired-end reads. In total, 451 million read pairs were generated, with yield, proportion aligned, error rate, intensity, and GC content provided in Table 1. RNA-Seq reads are available from the NCBI as Bioproject PRJNA342864, run accessions SRR4279903 and SRR4279904. Summary statistics for data yield, percent pass-filter (%PF), raw cluster percentage per lane, and quality score summary are provided in Supplementary Table S1. Filtering of poor quality reads (Q < 30) removed 32% of the original 451 million read pairs, with 305 million high-quality paired reads used for assembly. FastQC reports for raw, filtered and *in silico* normalized data are provided in Supplementary Figure S1.

**Table 1:** Spiny mouse RNA-Seq summary statistics.

	Forward	Reverse	Total
Yield total (Gb)	45.9	45.9	91.77617
Aligned (%)	0.45	0.25	0.3509741
Error rate (%)	0.62	1.6	1.003602
Intensity cycle 1	3255	3054	3154.379
%>=Q30	80.5	46.5	63.47869
Total raw read pairs			451,182,406
Total read pairs (Q> = 30)			305,920,540
GC content			45%

### *De novo* transcriptome assembly

A detailed protocol describing the assembly and validation of the spiny mouse RNA-Seq dataset is available at https://dx.doi.org/10.17504/protocols.io.ghebt3e. This protocol provides a brief description, documentation, citations, dependencies/requirements, parameters and commands used for all software employed in this manuscript.

**Figure 1:**
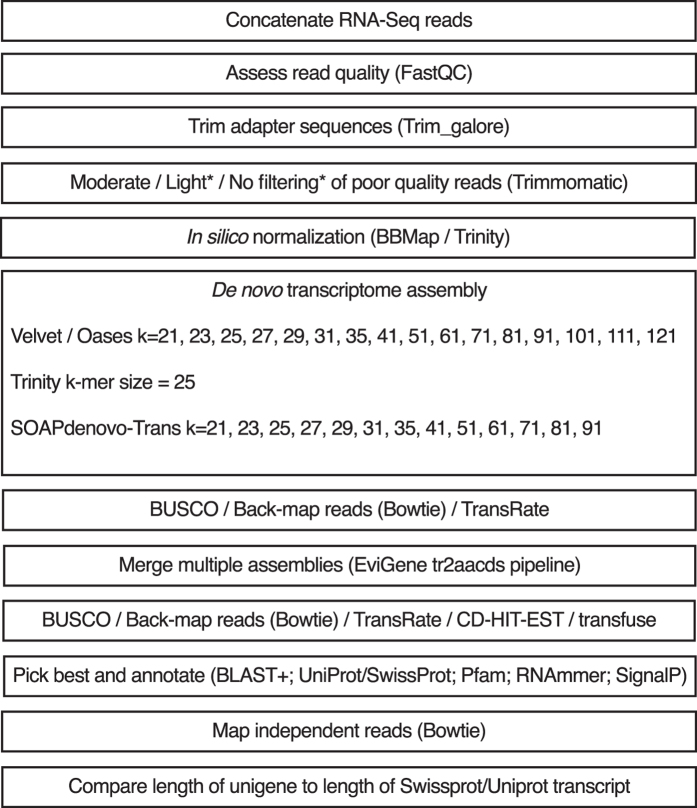
Flow chart of transcriptome assembly pipeline. *SEECER probabilistic error correction conducted on these datasets.

**Figure 2:**
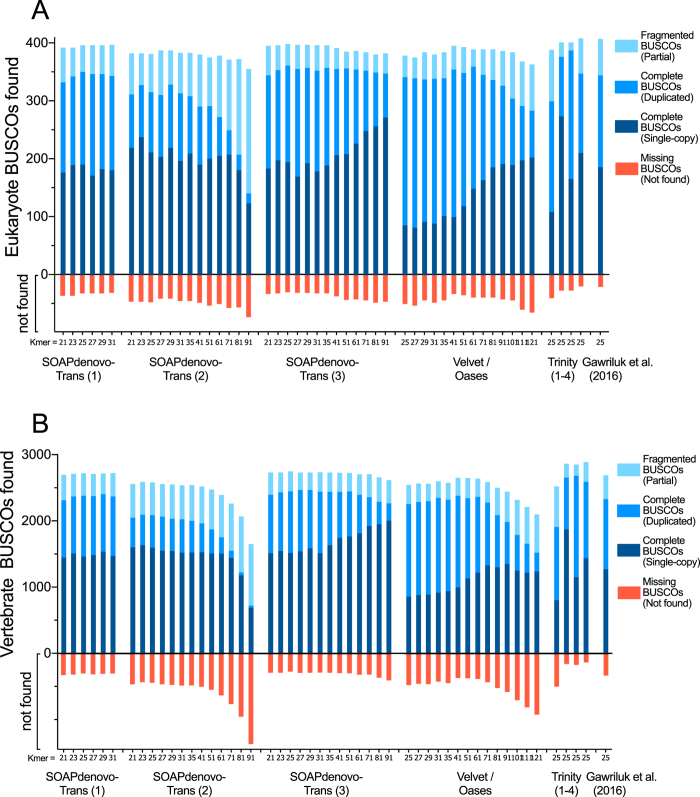
BUSCOs identified in each single-kmer transcriptome assembly. SOAPdenovo-Trans (1): reads prior to quality filtering; SOAPdenovo-Trans (2): light filtering of poor quality read pairs, combined with *in silico* normalization; SOAPdenovo-Trans (3): moderate filtering of poor quality read pairs; Velvet/Oases: moderate filtering of poor quality read pairs; Trinity (1): light filtering of poor quality read pairs, combined with *in silico* normalization; Trinity (2): light filtering of poor quality read pairs, combined with SEECER error correction; Trinity (3): moderate filtering of poor quality read pairs; Trinity (4): light filtering of poor quality read pairs, assembled with ver2.3.2.

**Figure 3:**
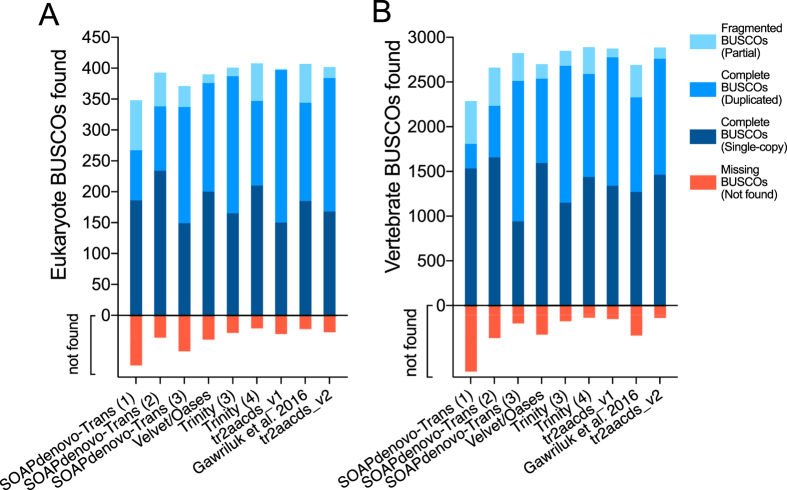
BUSCOs identified in each clustered/multi-kmer transcriptome assembly. SOAPdenovo-Trans (1): no filtering; (2): light filtering of poor quality read pairs, combined with *in silico* normalization; (3): moderate filtering of poor quality read pairs; Velvet/Oases: moderate filtering of poor quality read pairs; Trinity (1): light filtering of poor quality read pairs, combined with *in silico* normalization; (2): light filtering of poor quality read pairs, combined with SEECER error correction; (3): moderate filtering of poor quality read pairs; (4) moderate filtering of poor quality reads and SEECER error correction; tr2aacds merged assemblies (1): pre-v2.3.2 Trinity; (2) including v2.3.2 Trinity.

**Figure 4:**
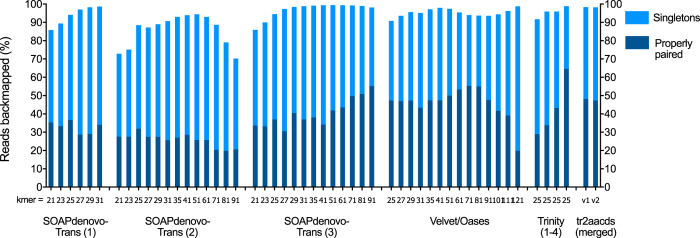
Proportion of reads backmapping to each transcriptome assembly. SOAPdenovo-Trans (1): no filtering; (2): light filtering of poor quality read pairs, combined with *in silico* normalization; (3): moderate filtering of poor quality read pairs; Velvet/Oases: moderate filtering of poor quality read pairs; Trinity (1): light filtering of poor quality read pairs, combined with *in silico* normalization; (2): light filtering of poor quality read pairs, combined with SEECER error correction; (3): moderate filtering of poor quality read pairs; (4): moderate filtering of poor quality reads and SEECER error correction; tr2aacds merged assemblies (1): pre-v2.3.2 Trinity; (2): including v2.3.2 Trinity.

**Figure 5:**
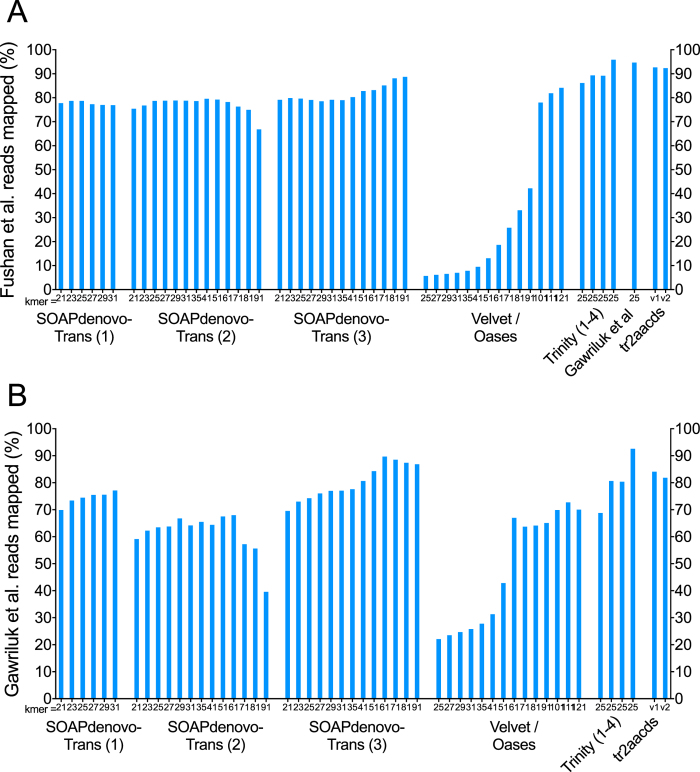
Proportion of independent reads mapping to each transcriptome assembly for (**A**) PRJNA184055 (Fushan *et al*., 2015), and (**B**) PRJNA300275 (Gawriluk *et al*., 2016). SOAPdenovo-Trans (1): no filtering; (2): light filtering of poor quality read pairs, combined with *in silico* normalization; (3): moderate filtering of poor quality read pairs; Velvet/Oases: moderate filtering of poor quality read pairs; Trinity (1): light filtering of poor quality read pairs, combined with in silico normalization; (2): light filtering of poor quality read pairs, combined with SEECER error correction; (3): moderate filtering of poor quality read pairs; (4): moderate filtering of poor quality reads and SEECER error correction; (5): Gawriluk *et al*. transcriptome assembly; tr2aacds merged assemblies (1): pre-v2.3.2 Trinity; (2): including v2.3.2 Trinity.

**Figure 6:**
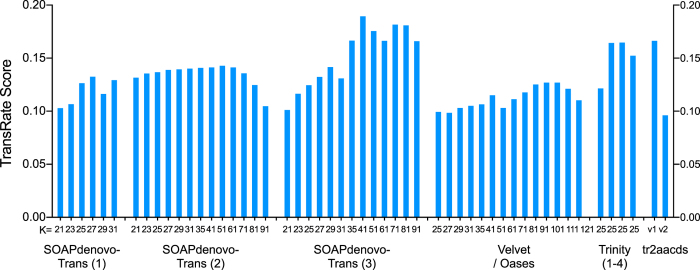
TransRate scores for each transcriptome assembly. SOAPdenovo-Trans (1): no filtering; (2): light filtering of poor quality read pairs, combined with *in silico* normalization; (3): moderate filtering of poor quality read pairs; Velvet/Oases: moderate filtering of poor quality read pairs; Trinity (1): light filtering of poor quality read pairs, combined with *in silico* normalization; (2): moderate filtering of poor quality read pairs; (3): light filtering of poor quality read pairs, combined with SEECER error correction; (4) Trinity v2.3.2 after moderate filtering of poor quality read pairs; (5) Gawriluk *et al*. transcriptome assembly; tr2aacds merged assemblies (1): pre-v2.3.2 Trinity; (2) including v2.3.2 Trinity.

**Figure 7:**
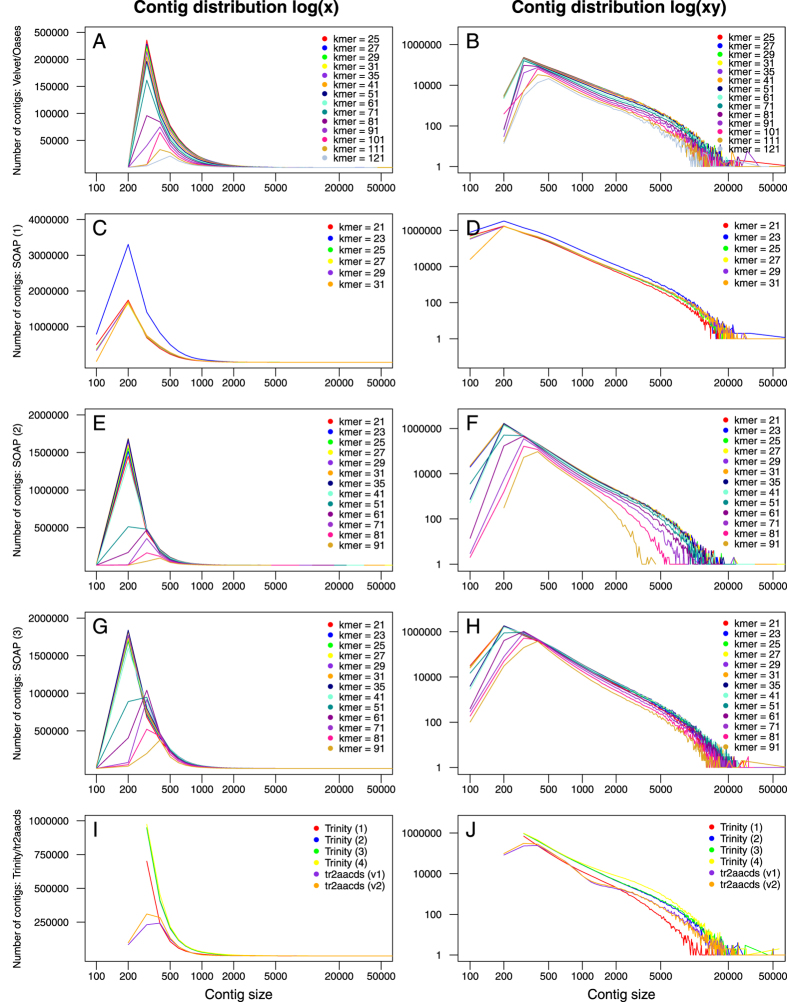
Distribution of contig number and size for all assemblies. Velvet/Oases: moderate filtering of poor quality read pairs; SOAPdenovo-Trans (1): no filtering; (2): light filtering of poor quality read pairs, combined with *in silico* normalization; (3): moderate filtering of poor quality read pairs; Trinity (1): light filtering of poor quality read pairs, combined with *in silico* normalization; (2): moderate filtering of poor quality read pairs; (3): light filtering of poor quality read pairs, combined with SEECER error correction; (4) Trinity v2.3.2 after moderate filtering of poor quality read pairs; tr2aacds merged assemblies (v1): pre-v2.3.2 Trinity; (v2) including v2.3.2 Trinity output.

In total, 50 unique single-kmer transcriptome assemblies were produced from 305 million paired reads, with and without digital normalization and read error correction, as described in Fig. 1. Detailed metrics for all transcriptome assemblies are provided in Supplementary Table S2. Digital normalization using Trinity reduced the size of the dataset by >80%, however assemblies constructed using normalized data contained fewer ‘Benchmarking Universal Single-Copy Orthologs’ (BUSCOs)^13^ (Figs 2 and 3), had decreased backmapping rates (‘backmapping’ is aligning the reads used for *de novo* assembly to the assembled transcripts) (Fig. 4), decreased mapping of independent spiny mouse read data (Fig. 5), and worse TransRate scores^14^ (Fig. 6), compared to assemblies generated from unnormalized data. Size distribution of assembled transcripts comprising each assembly is described in Fig. 7.

Clustering highly similar Trinity^15^ contiguous sequences (contigs) using CD-HIT-EST^16, 17^ resulted in a modest reduction in the total number of Trinity contigs, with the majority of clustered contigs corresponding to transcript isoforms (Supplementary Figure S2). Clustering highly similar contigs increased the proportion of single copy BUSCO orthologs detected, however it also increased the number of fragmented BUSCO orthologs (Supplementary Figure S3).

Read sequencing errors identified using probabilistic error correction program SEECER^18^ were comprised of 14,821,705 substitutions (4.84%), 1,760,162 deletions (0.57%), and 1,614,908 insertions (0.53%), affecting 6% of reads in total. Error correction provided a modest improvement to BUSCO score (Figs 2 and 3) and mapping of independent reads when assembled using Trinity (Fig. 4), however it also resulted in slightly poorer backmapping rate and TransRate score, compared to assemblies generated from non-corrected reads (Figs 5 and 6).

**Figure 8:**
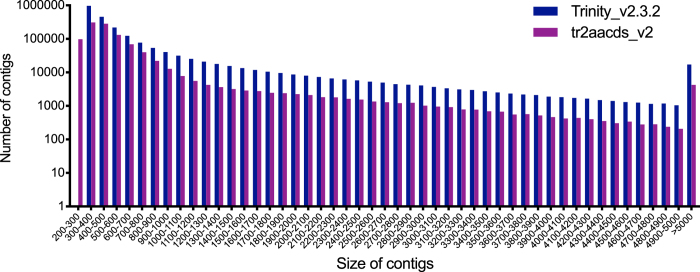
Comparison of contig size and number for Trinity_v2.3.2 (moderate filtering of poor quality read pairs) and tr2aacds_v2 (including v2.3.2 Trinity output) assemblies.

**Figure 9:**
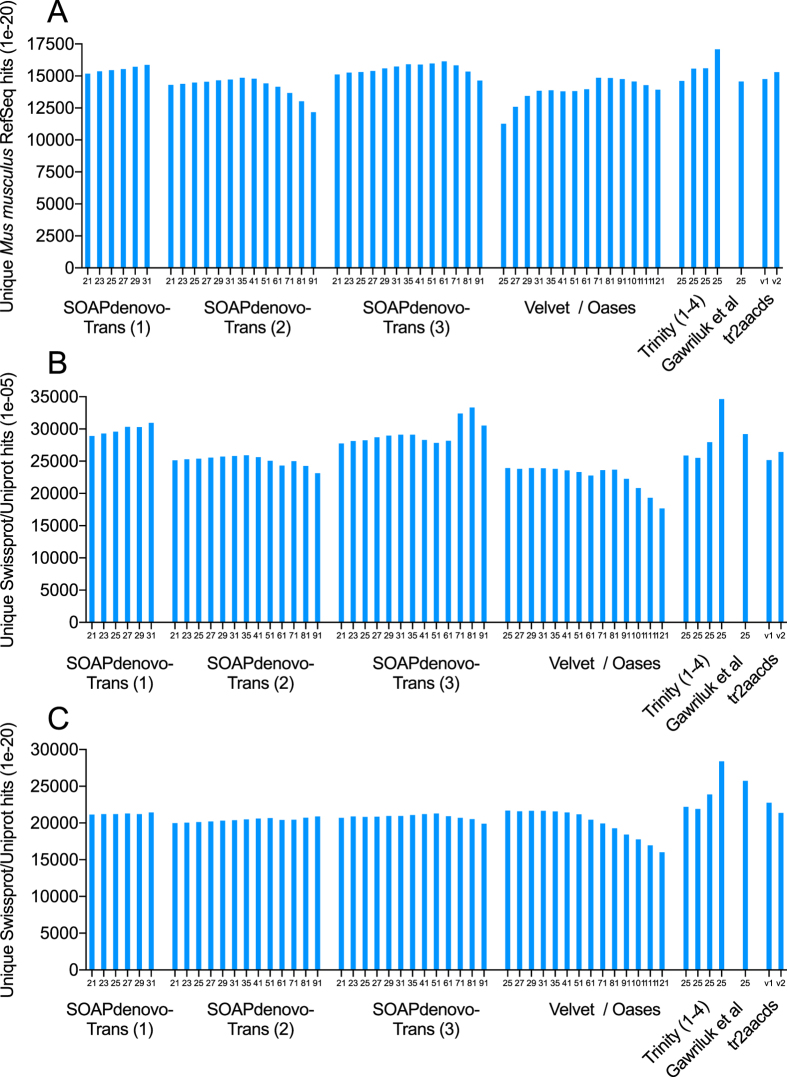
Unique BLAST hits for each assembly when aligned to: (**A**) *Mus musculus* RefSeq transcript database (e-value ≤ 1 × 10^−20^), (**B**) UniProtKB/SwissProt database (e-value ≤ 1 × 10^−5^), (**C**) UniProtKB/SwissProt database (e-value ≤ 1 × 10^−20^). SOAPdenovo-Trans (1): no filtering; (2): light filtering of poor quality read pairs, combined with *in silico* normalization; (3): moderate filtering of poor quality read pairs; Trinity (1): light filtering of poor quality read pairs, combined with *in silico* normalization; (2): moderate filtering of poor quality read pairs; (3): light filtering of poor quality read pairs, combined with SEECER error correction; (4) Trinity v2.3.2 after moderate filtering of poor quality read pairs; tr2aacds merged assemblies (v1): pre-v2.3.2 Trinity; (v2) including v2.3.2 Trinity output.

**Figure 10:**
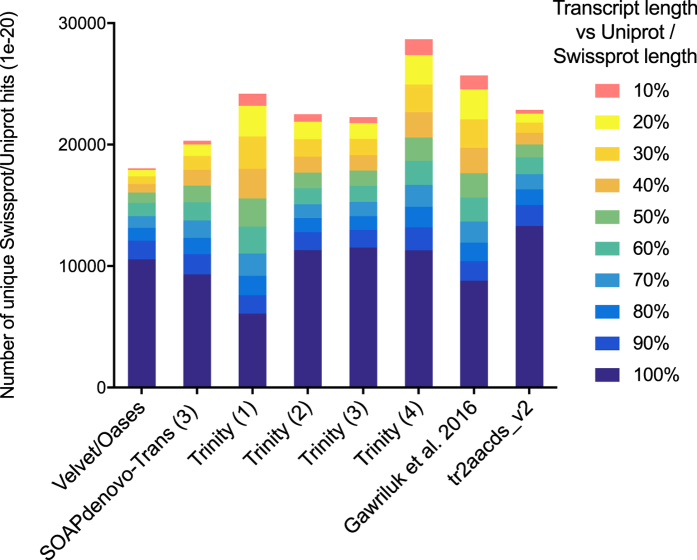
Assessing the number of full-length protein-coding transcripts within transcriptome assemblies, with UniProtKB/SwissProt BLAST hits (e-value ≤ 1 × 10^−20^) categorised by length of alignment (nucleotides) with the UniProtKB/SwissProt reference transcript. Transcripts are considered ‘full-length’ if they are >90% of the reference transcript length. SOAPdenovo-Trans (3): moderate filtering of poor quality read pairs; Trinity (1): light filtering of poor quality read pairs, combined with *in silico* normalization; (2): moderate filtering of poor quality read pairs; (3): light filtering of poor quality read pairs, combined with SEECER error correction; (4) Trinity v2.3.2 after moderate filtering of poor quality read pairs; tr2aacds merged assemblies (v1): pre-v2.3.2 Trinity; (v2) including v2.3.2 Trinity output.

**Figure 11:**
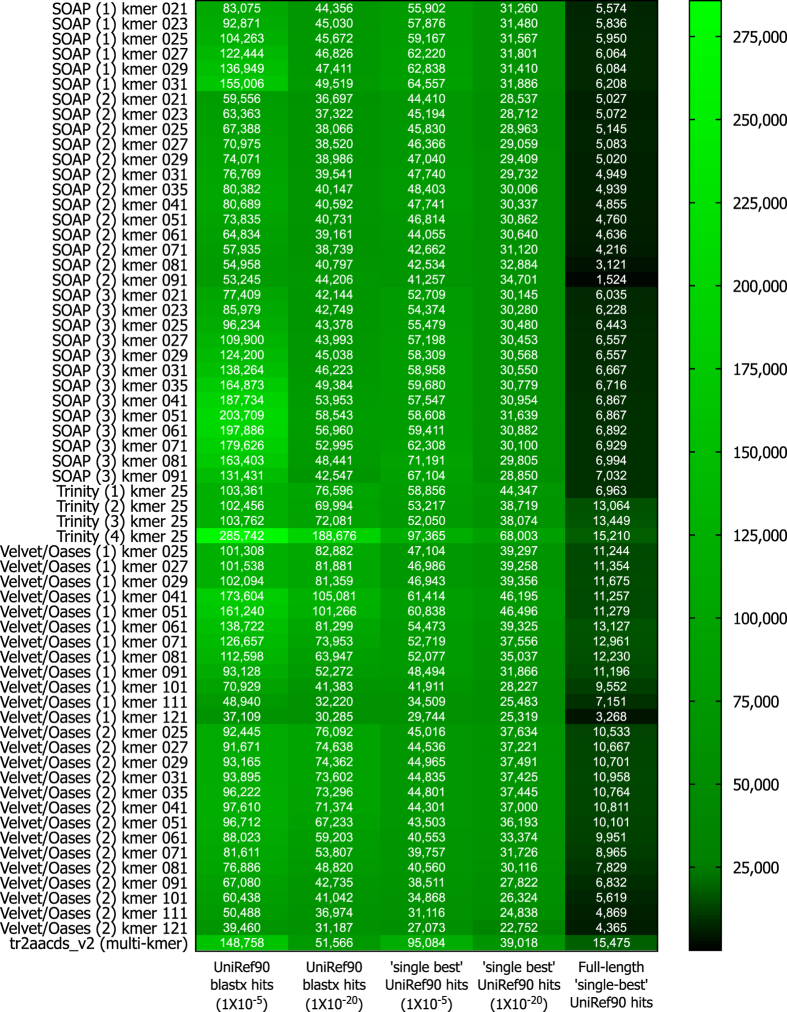
Diamond BLASTx hits from the UniRef90 database. SOAPdenovo-Trans (1): no filtering; (2): light filtering of poor quality read pairs, combined with *in silico* normalization; (3): moderate filtering of poor quality read pairs; Trinity (1): light filtering of poor quality read pairs, combined with *in silico* normalization; (2): moderate filtering of poor quality read pairs; (3): light filtering of poor quality read pairs, combined with SEECER error correction; (4) Trinity v2.3.2 after moderate filtering of poor quality read pairs; Velvet/Oases (1): moderate filtering of poor quality read pairs; Velvet/Oases (2): moderate filtering of poor quality read pairs combined with *in silico* normalization.

Trinity produced the largest and most complete single-kmer assemblies. The Trinity_v2.3.2 assembly contained the greatest number of BUSCOs (Figs 2 and 3), the highest proportion of back-mapped reads (Fig. 4), and the highest proportion of aligned RNA-Seq reads from the National Center for Biotechnology Information (NCBI) projects PRJNA184055 and PRJNA292021 (Fig. 5). The distribution of contigs is more negatively skewed, with more contigs of larger size (Figs 7 and 8). The Trinity_v2.3.2 assembly also has the highest number of ‘Basic Local Alignment Search Tool’ (BLAST) hits, and unique ‘single best’ BLAST hits (the single highest scoring assembled transcript alignment for each database entry above a specified significance threshold), compared to *Mus musculus* RefSeq transcripts, UniProtKB/SwissProt database^19, 20^ and UniRef90 database^21, 22^ (Figs 9, 10 and 11). Gene Ontology (GO) terms corresponding to annotated transcripts were well distributed between the categories of biological process, cellular component and molecular function (Supplementary Figure S4).

### Collating non-redundant transcripts from multiple assemblies

Merging non-redundant transcripts from all assemblies using the EvidentialGene ‘transcript to amino acid coding sequence’ (*tr2aacds*) pipeline^23, 24^ (Supplementary Table S2) increased the proportion of complete BUSCOs found, and reduced the number of fragmented and missing BUSCOs (Fig. 3). The BUSCO values obtained are consistent with the most complete reference transcriptomes from other vertebrate and eukaryote taxa (BUSCO^13^: Supplementary Online Material).

### Annotation and identification of non-coding RNAs

The most accurate and complete single-kmer assembly was produced by Trinity_v2.3.2 from the ‘non-normalized’ dataset. It contains 2,219,978 contigs (2,026,183 ‘genes’ as defined by Trinity), representing a 1.29 Gb transcriptome. Of these, 546,398 transcripts were identified as non-coding by the ‘Coding-Non-Coding Index’ (CNCI^25^) corresponding to 44,572 unique NONCODE ncRNAs^26^, 277,565 transcripts aligned to UniProtKB/SwissProt entries (BLASTx, ‘Expect value’ (e-value) ≤ 1 × 10^−20^), and 145,658 transcripts contained an open reading frame (ORF). Many UniProtKB/SwissProt entries have more than one aligning transcript from the assembly: the highest scoring transcript for each UniProtKB/SwissProt entry (the ‘single best’ BLAST hit) was identified and collated. In total, 28,847 unique ‘single best’ alignments to the UniProtKB/SwissProt database were identified, 21,762 of which contain an ORF. In comparison, the *tr2aacds*-generated assembly (“tr2aacds_v2”) contains fewer than half the number of transcripts compared to the Trinity_v2.3.2 assembly, with 1,034,437 transcripts in total, representing a 491 Mb transcriptome. Of these, 258,400 transcripts were identified as non-coding by CNCI corresponding to 31,642 unique NONCODE ncRNAs, 135,139 transcripts show significant sequence similarity to the UniProtKB/SwissProt database (e-value ≤ 1 × 10^−20^), and 117,250 transcripts contain an ORF. Alignment to the UniProtKB/SwissProt database revealed 22,852 unique ‘single best’ transcripts, 21,135 of which contain an ORF.

Despite containing fewer ‘single best’ UniProtKB/SwissProt-aligning transcripts, analysis of single best transcripts revealed the tr2aacds_v2 assembly contains the greatest number of full-length transcripts (transcript length >90% of the reference UniProtKB/SwissProt entry) (Fig. 10). Combined with a greater number of complete BUSCOs, and fewer fragmented BUSCOs, this result suggests the tr2aacds_v2 assembly is the most accurate and complete catalogue of protein-coding transcripts for the spiny mouse.

## Discussion

The transcriptome assemblies produced and validated here comprise an important new resource for spiny mouse research, increasing the value and accessibility of this species as an animal model in biomedical science. In total, 50 assemblies were produced using three *de novo* assemblers: Trinity^15^, SOAPdenovo-Trans^27^, and Oases^28^. Combining unique assembled transcripts from all single-kmer assemblies using EvidentialGene *tr2aacds* produced the largest collection of full-length protein-coding transcripts. Each transcriptome performed well in measures of assembly integrity and completeness (e.g. BUSCO, TransRate, backmapping and BLAST^29, 30^), however TransRate scores were lower than expected for high-quality *de novo* transcriptome assemblies.

The approximate median TransRate score for assemblies uploaded to the NCBI Transcriptome Shotgun Assembly database is 0.2, however the best scoring assemblies were between 0.15 and 0.2. Potential explanations for the lower-than-expected TransRate scores are the proportion of read errors identified by SEECER, and quality of the RNA-Seq reads. TransRate scoring is contingent on accurate alignment of reads to transcripts, as it evaluates assemblies based on whether each base has been called correctly, whether bases are truly part of transcripts, whether contigs are derived from a single transcript, and whether contigs are structurally complete and correct^14^. Alignment rates calculated by TransRate were below the alignment rates expected (based on read alignment using Bowtie), and this may have negatively impacted the TransRate score. Using error-corrected reads for TransRate alignment may increase TransRate scores compared to uncorrected reads^31^, however this was not examined in the present study.

The largest catalogue of ‘full length’ (>90%) transcripts aligning to the UniProtKB/SwissProt database was produced using the EvidentialGene *tr2aacds* pipeline. This finding correlates with similar projects incorporating *tr2aacds*-based meta-assembly, with more accurate and complete gene sets produced compared to transcriptomes assembled with a single software package (for instance, the mosquito *Aedes aegypti*: http://arthropods.eugenes.org/EvidentialGene/arthropods/mosquito/aedes_aegypti/). Generating accurate and complete transcripts is fundamental for gene annotation, and for subsequent identification of gene function, however a transcriptome assembly is comprised of more than protein-coding transcripts alone. Non-coding transcripts such as micro-RNAs and long non-coding RNAs perform essential roles in cellular function, with novel investigative methodologies driving increased interest in this area. The Trinity_v2.3.2 assembly contained the largest number of non-coding transcripts of all assemblies produced, and this resource will be made available in addition to the tr2aacds_v2 assembly.

A fundamental goal in generating this dataset is to facilitate access to spiny mouse transcript sequence information for external collaborators and researchers. The sequence reads and metadata are available from the NCBI (PRJNA342864) and assembled transcriptomes (Trinity_v2.3.2 and tr2aacds_v2) are available from the Zenodo repository (https://doi.org/10.5281/zenodo.808870), however accessing and utilizing this data can be challenging for researchers lacking bioinformatics expertise. To address this problem we are hosting a SequenceServer^32^ BLAST-search website (http://spinymouse.erc.monash.edu/sequenceserver/). This resource provides a user-friendly interface to access sequence information from the tr2aacds_v2 assembly (to explore annotated protein-coding transcripts) and/or the Trinity_v2.3.2 assembly (to explore non-coding transcripts).

The public spiny mouse BLAST database has already been used by the spiny mouse research community. One member of our research group has used this resource to successfully design quantitative real-time polymerase chain reaction (qPCR) primer sets for lung-specific genes, markers of hypoxia, inflammation and apoptosis, and nuclear coding genes associated with mitogenesis (n = 41; personal communication). Sanger sequencing of PCR products confirmed >85% success under standard qPCR conditions, which is a significant improvement compared to ~40% success rate reported for primer design based on homologous regions from human, mouse (*Mus musculus*) and rat (*Rattus norvegicus*)^33–35^. Another collaborator requested the sequence of the spiny mouse protein-coding Beta Amyloid transcript, the product of which is implicated in the etiology of Alzheimer’s disease. Prior to release of our dataset, the Gawriluk *et al*. transcriptome assembly was the only potential source for this information, however a complete Beta Amyloid transcript was not found in the assembly. The complete transcript from our database shows biological variation between the spiny mouse, *Mus musculus* (NM_001198823.1), and human orthologs (M15532.1).

RNA-Seq provides an unprecedented opportunity for cost-effective, large-scale genetic analysis in non-model organisms for which a genome sequence is unavailable. *De novo* assembly of millions/billions of RNA-Seq reads into a reference transcriptome can provide a valuable scientific resource, with applications in phylogenetics^36^, novel gene identification^37^, RNA editing^38^ and alternative splicing investigation^39^, qPCR primer design^40^, development and refinement of bioinformatics software^41^, augmenting proteomic research^42^, and investigation of gene expression profiles underlying complex physiological traits^43, 44^. The utility of a transcriptome assembly is highly dependent on its completeness and accuracy, however there is not yet consensus within the transcriptomics community on a universal ‘gold-standard’ protocol or quality metric for transcriptome assembly. Many popular *de novo* assembly software packages provide detailed protocols for their use (eg. Trinity^45^), and practical guidelines such as the comprehensive Oyster River protocol^31^ provide clear advice and guidance for optimising *de novo* assembly (http://oyster-river-protocol.readthedocs.io/en/latest/), however substantial customization and optimization of the sequencing and assembly pipeline is often necessary to produce high-quality, meaningful results^46, 47^. The transcriptome assembly pipeline optimised for this project is easily accessible (https://dx.doi.org/10.17504/protocols.io.ghebt3e), facilitating reproduction and replication in other species.

## Conclusion

In conclusion, we have generated the most accurate and complete *de novo* transcriptome for the spiny mouse (*Acomys cahirinus*) to date, using the combined output of three *de novo* transcriptome assemblers: Trinity, SOAPdenovo-Trans, and Oases. All assemblies produced (n = 50) were analysed for accuracy and completeness, and validated using multiple quality metrics. The highest quality single-kmer transcriptome was generated using Trinity (v2.3.2). It is comprised of 2,219,978 transcripts, representing a 1.29 Gb transcriptome. The EvidentialGene *tr2aacds* pipeline was effective in identifying and collating unique transcripts from all 50 assemblies, producing a 491 Mb transcriptome comprised of 1,034,437 transcripts. This meta-assembly contained a greater number of full-length protein-coding transcripts than all individual single-kmer assemblies. This is the first study to implement the EvidentialGene *tr2aacds* pipeline to augment transcriptome assembly in a mammal. This study has produced the largest gene catalogue to date for the spiny mouse, providing an important resource for medical research. This dataset is now being used to further investigate physiological traits unique to the spiny mouse.

## Methods

### Data processing

The protocol used to assemble and validate the spiny mouse RNA-Seq dataset is available at https://dx.doi.org/10.17504/protocols.io.ghebt3e.

Sequence reads were quality checked using FastQC v0.11.3 (http://www.bioinformatics.babraham.ac.uk/projects/fastqc/). Adapter sequences and low quality bases (Q < 20) were trimmed from 3′ ends using trim-galore (ver: 0.4.0; http://www.bioinformatics.babraham.ac.uk/projects/trim_galore/), which implements cutadapt v0.9.5^48^. Reads with average quality scores lower than 20 and read pairs in which either forward or reverse reads were trimmed to fewer than 35 nucleotides were discarded. Remaining reads were assessed again using FastQC, to ensure adapter sequences are eliminated.

Further filtering of poor quality reads was conducted using Trimmomatic v0.30^49^ with settings "LEADING:3 TRAILING:3 SLIDINGWINDOW:4:20 AVGQUAL:30 MINLEN:35”. Nucleotides with quality scores lower than 3 were trimmed from the 3′ and 5′ read ends. Reads with an average quality score lower than 30, and reads with a total length of fewer than 35 nucleotides after trimming were removed. Probabilistic error correction was performed on trimmed/filtered reads using SEECER^18^ with default parameters. Both corrected and uncorrected reads were subjected to *de novo* assembly.

### *De novo* transcriptome assembly

Reads were assembled using either SOAPdenovo-Trans v1.03^27^, Trinity^15^ package r20140413p1 and Trinity package v2.3.2 (available at https://sourceforge.net/projects/trinityrnaseq/files/PREV_CONTENTS/previous_releases/ and http://trinityrnaseq.github.io), or Velvet v1.2.10^50^/Oases v0.2.08^28^ with default parameters, except where indicated. The single-kmer assemblies were performed with and without digital normalization and error correction as described in Fig. 1. Reads were subjected to digital normalization using the “--normalize_reads” feature in Trinity.

SOAPdenovo-Trans parameters: “max_rd_len = 150, rd_len_cutof = 150, avg_ins = 192, reverse_seq = 0, asm_flags = 3” with kmer lengths: 21, 23, 25, 27, 29, 31, 35, 41, 51, 61, 71, 81, 91. Trinity was used at kmer length 25, with parameters: “--normalize_reads --seqType fq --JM 100 G --CPU 20 --min_kmer_cov 2”. Reads were assembled with Velvet at kmer lengths 21, 23, 25, 27, 29, 31, 35, 41, 51, 61, 71, 81, 91, 101, 111, 121. Velvet was compiled with parameters “MAXKMERLENGTH = 141 BIGASSEMBLY = 1 LONGSEQUENCES = 1 OPENMP = 1”. Velveth was run using “25,33,2 -shortPaired -fastq -separate”, “35 -shortPaired -fastq –separate” and “41,131,10 -shortPaired -fastq -separate”. Insert lengths of the fragments were estimated with CollectInsertSizeMetrics in Picard Tools version 1.90 (http://broadinstitute.github.io/picard/). Velvetg was run with parameters “-read_trkg yes -ins_length 215”. Oases was run with parameters “-min_trans_lgth 100 -ins_length 215”.

Assembly statistics were computed using the TrinityStats.pl from the Trinity package, and summary statistics are provided in log files produced by SOAPdenovo-Trans and Oases (Supplementary dataset 1).

### Collating non-redundant transcripts from multiple assemblies

The *tr2aacds* pipeline from the EvidentialGene package was used to identify and collate non-redundant transcripts from each individual transcriptome assembly. The *tr2aacds* pipeline predicts amino acid sequences and transcript coding sequences, removes transcript redundancy based on coding potential, removes sequence fragments, clusters highly similar sequences together into loci, and classifies non-redundant transcripts as ‘primary’ or ‘alternative’. Transcripts that scored poorly were removed, with remaining ‘primary’ and ‘alternative’ transcripts from each single-kmer assembly merged. This process was conducted twice: first (“tr2aacds_v1”) with all SOAPdenovo-Trans, Velvet/Oases, and Trinity r20140413p1 assemblies (“Trinity 1–3”), and again to incorporate the Trinity v2.3.2 assembly to create “tr2aacds_v2”.

Accuracy and completeness was assessed in all assemblies (single-kmer and tr2aacds) using BUSCO v1.1b1^13^ to establish the presence or absence of universal single copy orthologs common to vertebrates and eukaryotes. Accuracy was assessed by the proportion of original sequence reads mapped (‘backmapping’) to each assembly using Bowtie v0.12.9^51^ with settings: ‘-q --phred33-quals -n 2 -e 99999999 -l 25 -I 1-X 1000 -p 12 -a -m 200 --chunkmbs 256’. Independent RNA-Seq reads were obtained from the NCBI sequence read archive (SRA): datasets SRR636836, SRR636837, and SRR636838 were obtained from project PRJNA184055, and datasets SRR2146799–SRR2146807 from project PRJNA292021. These reads were generated from liver^10^ and skin^11^ and neither tissue was subjected to treatment - they are ‘control’ groups in their corresponding experiments. The independent RNA-Seq reads were aligned using Bowtie to each draft transcriptome assembly, with settings as specified above. The proportion of mapped reads was calculated using samtools flagstat with default parameters^52^. Structural integrity was examined using TransRate v1.0.3^14^ with default settings, in which Salmon v0.6.0^53, 54^ and SNAP-aligner v1.0 18beta^55^ were implemented. Redundancy in assembled transcripts was assessed by the proportion of highly similar contiguous sequences (contigs), clustered using CD-HIT-EST v4.6.5^16,17^ with settings ‘-c 0.95 -n 8 -p 1 -g 1 -M 200000 -T 8 -d 40’. Further clustering at 90%, 95%, and 100% similarity was conducted on a representative single-kmer assembly “Trinity (2)” to assess contig redundancy.

### Annotation and identification of non-coding RNAs

The best performing assembly was annotated using the Trinotate pipeline (ver2.0.2, http://trinotate.github.io/). In brief, *de novo* transcripts were aligned against the UniProtKB/SwissProt database (ftp://ftp.uniprot.org/pub/databases/uniprot/current_release/knowledgebase/complete/uniprot_sprot.fasta.gz; accessed 7th January 2016) using NCBI BLAST + BLASTx (for nucleotide sequences) and BLASTp (for protein sequences)^56^. Transdecoder v2.0.1 (https://transdecoder.github.io/) was used to predict ORFs, with BLASTp performed using translations of predicted ORFs as the query and UniProtKB/SwissProt database as the target. HMMER v3.1b1 and Pfam v27 databases^57^ were used to predict protein domains. SignalP v4.1^58^ was used to predict signal peptides, and RNAmmer v1.2^59^ to predict rRNAs. Annotations were loaded into an SQL database (packaged with Trinity: Supplementary dataset 2). Gene Ontology (GO) terms linked to the UniProtKB/SwissProt entry for each BLAST hit were used for ontology annotation. GO functional classifications were summarised using the Web Gene Ontology (WEGO) annotation plot (http://wego.genomics.org.cn)^60^. *De novo* transcripts were also aligned to *Mus musculus* RefSeq RNA transcripts (ftp://ftp.ncbi.nlm.nih.gov/refseq/M_musculus/mRNA_Prot/mouse.1.rna.fna.gz; accessed 3rd Feb, 2017) using NCBI BLAST + BLASTn with settings ‘-num_threads 32 -max_target_seqs. 1 -evalue 1e-20 -outfmt 6’.

Alignment of transcripts from each assembly to the UniRef90 database was conducted using DIAMOND v0.8.36^61^. This program is significantly faster than NCBI BLAST + for aligning nucleotide sequences to a protein database (up to 20,000X increase in speed). ‘DIAMOND BLASTx’ was used instead of ‘BLAST+ BLASTx’ to offset the larger size of the database: UniRef90 = ~53 million sequences; UniProtKB/SwissProt database = ~550 thousand sequences. Default settings were used, with the addition of parameters ‘--sensitive -p 40 -k 1 -e 1e-05 -b 40 -c 1’.

Non-coding RNA analysis was conducted using the Coding-Non-Coding Index (CNCI) signature identification tool (version 2, Feb 28^th^ 2014, https://github.com/www-bioinfo-org/CNCI; 0fa252b) profiling adjoining nucleotide triplets and classifying transcripts as protein-coding or non-coding, independent of known annotations^62^. Non-coding transcripts were identified using the “vertebrate species model”, and a threshold cutoff of −0.05^63–65^.

Figures were produced using R software v3.3.2 and GraphPad Prism 7.

## Electronic supplementary material

Supplementary Figures and Tables

Supplementary dataset 1

Supplementary dataset 2
