# Supplementary material for: De novo transcriptome assembly for the spiny mouse (Acomys cahirinus)
Source: Sci Rep. 2017 Aug 21;7:8996. doi: 10.1038/s41598-017-09334-7 (PMC5566366; doi:10.1038/s41598-017-09334-7)
Supplement: Supplementary file 1 — Supplementary Figures and Tables [file 41598_2017_9334_MOESM1_ESM.pdf]

De novo transcriptome assembly for the spiny mouse (*Acomys cahirinus*)

Jared Mamrot, Roxane Legaie, Stacey J Ellery, Trevor Wilson, Torsten Seemann, David R. Powell, David K. Gardner, David W. Walker, Peter Temple-Smith, Anthony T. Papenfuss, Hayley Dickinson

Supplementary Figures

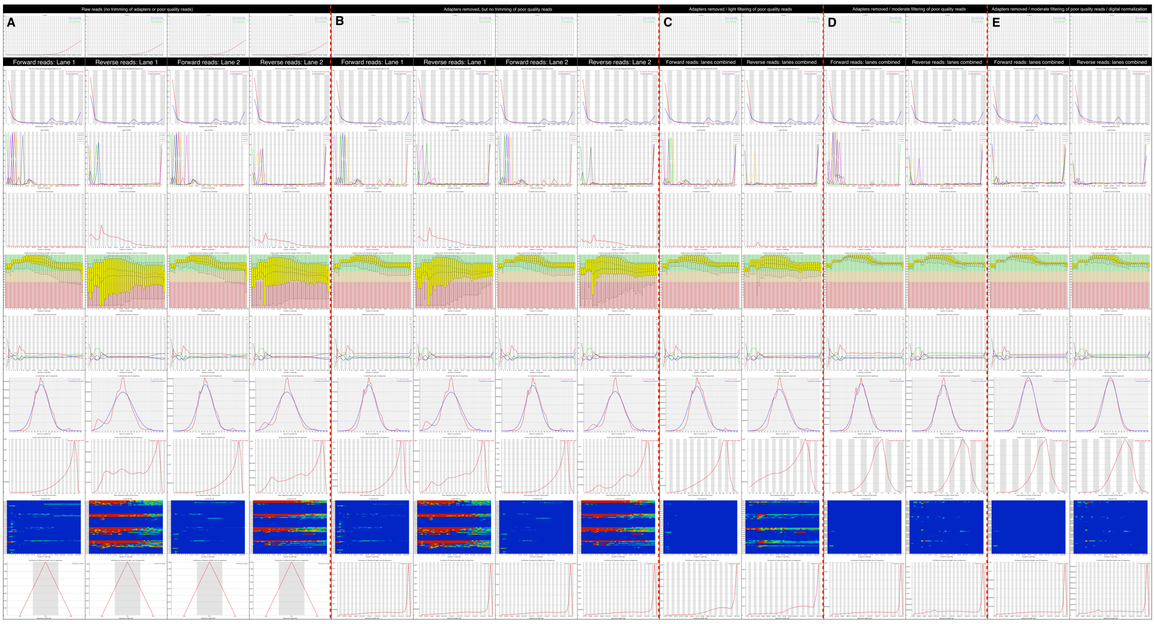

Supplementary Figure S1: FastQC reports for RNA-Seq reads: (A) unfiltered, (B) light filtering of poor quality reads, (C) moderate filtering of concatenated reads, (D) Strict quality filtering, (E) In silico normalization.

Full resolution image: https://doi.org/10.6084/m9.figshare.5170783.v1

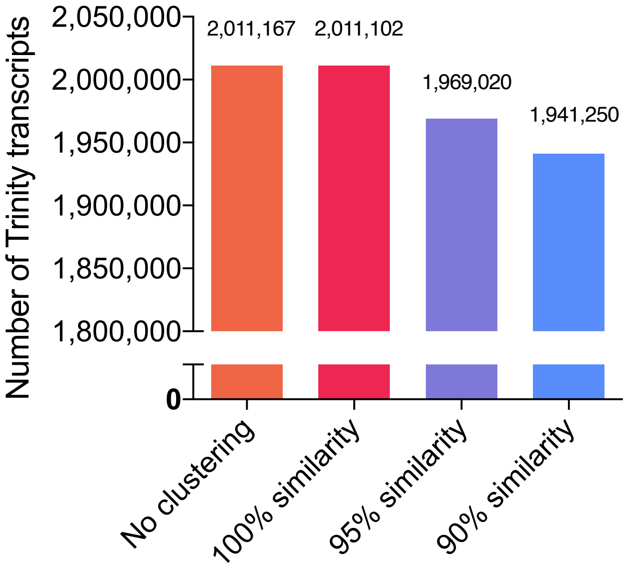

Supplementary Figure S2: Number of Trinity transcripts after CD-HIT-EST clustering at specified thresholds (100%, 95%, 90%).

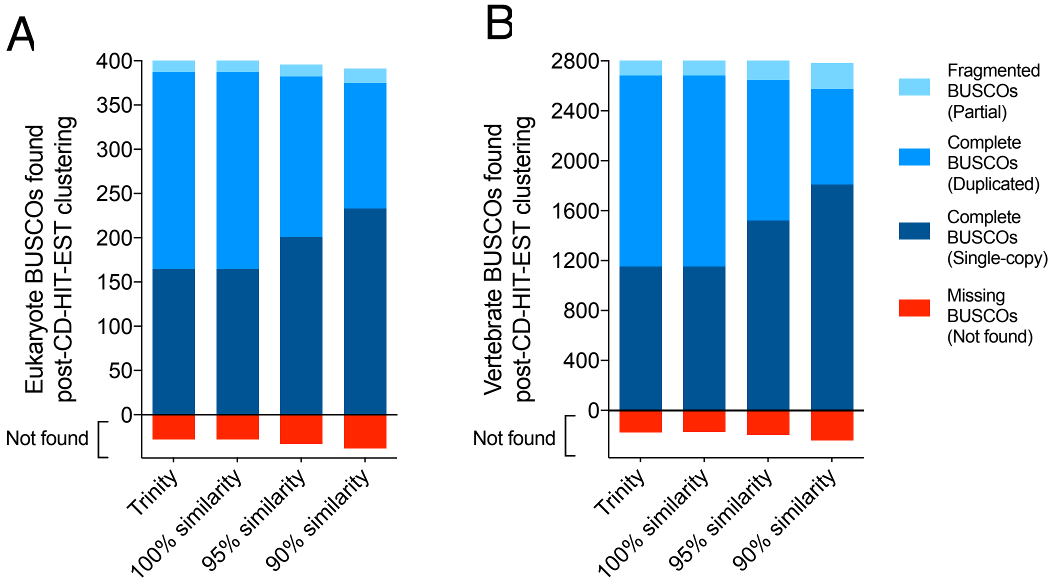

Supplementary Figure S3: BUSCO analysis after CD-HIT-EST clustering at specified thresholds (100%, 95%, 90%). (A) Eukaryota, (B) Vertebrata

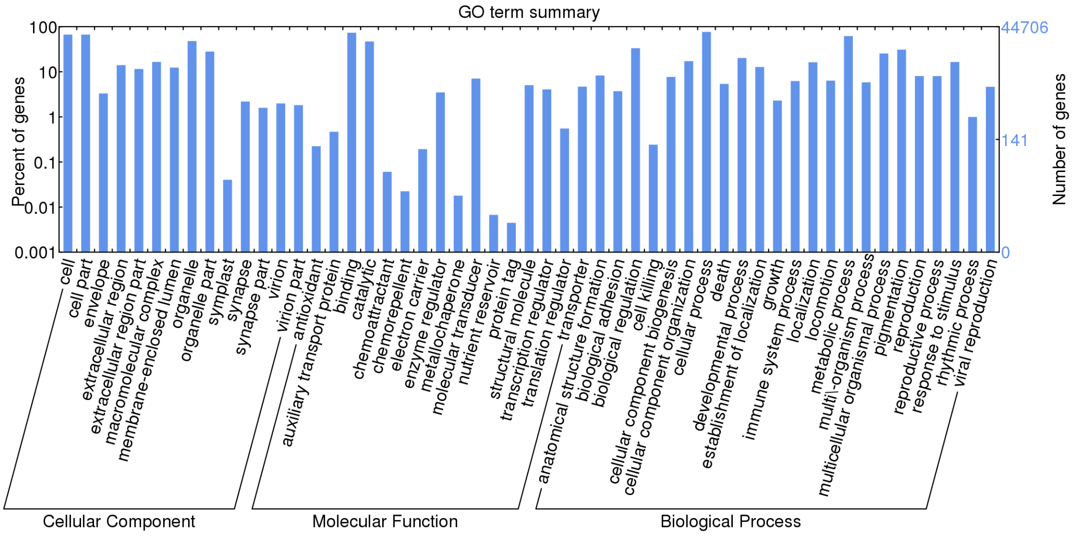

Supplementary Figure S4: Summary of gene ontology (GO) terms associated with SwissProt/UniProt blast hits.

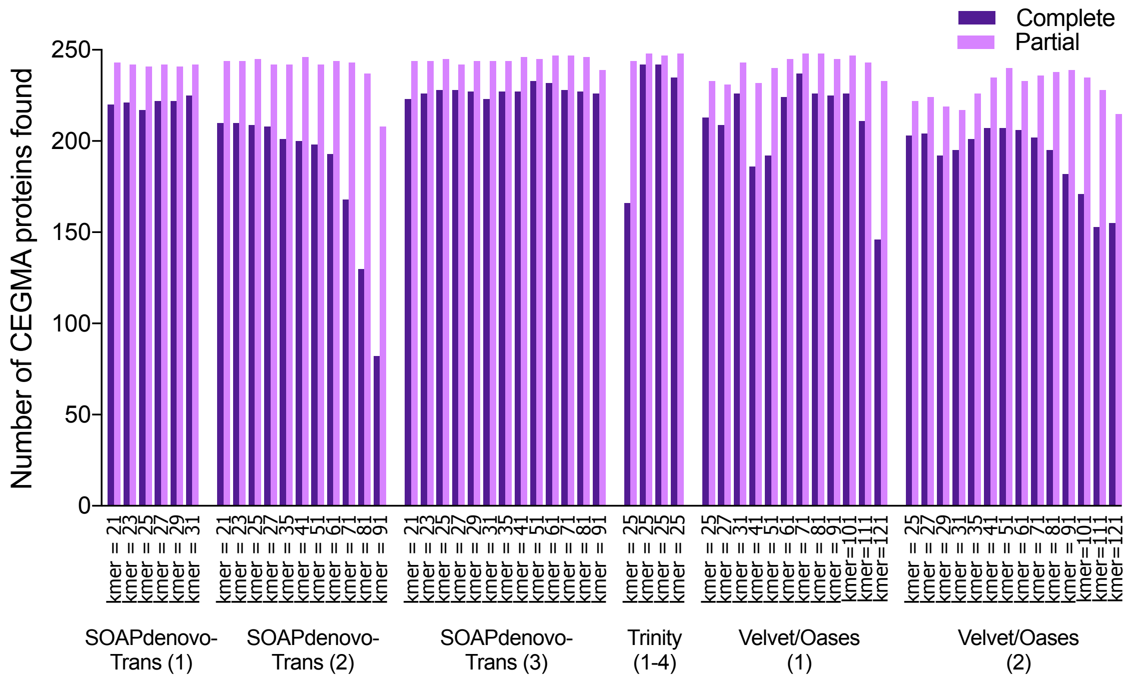

Supplementary Figure S5: Number of complete/partial CEGMA proteins found in each assembly.

Table S1. Summary statistics for RNASeq runs.

	Forward reads		Reverse reads		Total
Yield Total (G)	45.9		45.9		91.77617
Aligned (%)	0.45		0.25		0.3509741
Error Rate (%)	0.62		1.6		1.003602
Intensity Cycle 1	3255		3054		3154.379
% >= Q30	80.5		46.5		63.47869
Lane	1	2	1	2	
Tiles	64	64	64	64	
Density (K/mm2)	1209 ± 78	1242 ± 72	1209 ± 78	1242 ± 72	
Clusters PF (%)	64.69 ± 14	69.66 ± 10	64.69 ± 14	69.66 ± 10	
Phas/Prephas (%)	0.157 / 0.202	0.151 / 0.209	0.076 / 0.121	0.100 / 0.164	
Reads (M)	222.79	228.94	222.79	228.94	
Reads PF (M)	145.46	160.46	145.46	160.46	
% >= Q30	80.2	80.7	42.1	50.4	
Yield(G)	22	24	22	24	
Cycles Err Rated	150	150	0 150 0 150		
Aligned (%)	0.44 ± 0.06	0.46 ± 0.05	0.22 ± 0.20	0.28 ± 0.20	
Error Rate (%)	0.65 ± 0.30	0.59 ± 0.24	1.59 ± 0.51	1.61 ± 0.90	
Error Rate 35 cycle	0.33 ± 0.26	0.24 ± 0.21	0.89 ± 0.39	0.95 ± 0.61	
Error Rate 75 cycle	0.38 ± 0.27	0.31 ± 0.22	1.27 ± 0.51	1.18 ± 0.60	
Error Rate 100 cycle	0.44 ± 0.27	0.38 ± 0.29	1.35 ± 0.52	1.30 ± 0.69	
Intensity Cycle 1	3285 ± 266	3225 ± 264	2995 ± 1829	3113 ± 1678	

Supplementary Table S2: EvidentialGene tr2aacds pipeline output (class tables)

Class Table for Tr2aacds_v1

class	okay	drop	okay	drop
althi	11.2	1.2	112390	12098
althi1	6.1	1.5	61522	15135
althia2	0	0	0	629
altmfrag	0.2	0	2272	364
altmfraga2	0	0	171	21
altmid	0.8	0	8259	856
altmida2	0.1	0	1276	84
main	11.1	0.6	112092	6870
maina2	0.2	0	2865	629
noclass	58.5	6.1	587241	61278
noclassa2	0	0	372	98
parthi	0	1.2	0	12337
parthi1	0	0.3	0	3918
parthia2	0	0	0	511
total	88.5	11.4	888460	114828

AA-quality for okay set (no okalt): all and longest 1000 summary

	n	average	median	min,max	sum	gaps
okay.top	1000	2138	1837	1397, 29738	2138352	110, 0.1
okay.all	702570	100	83	40, 29738	70957577	224333, 0.3

Class Table for Tr2aacds_v2

class	okay	drop	okay	drop
althi	1.9	18.2	239423	2211673
althi1	1.1	17	133676	2065173
althia2	0	0.2	0	26395
altmfrag	0	0.2	3396	27243
altmfraga2	0	0	324	1285
altmid	0.1	1	16618	122236
altmida2	0	0.1	3081	13216
main	3.3	15.7	406187	1916052
maina2	0	0.5	10564	63529
noclass	1.8	29.1	221430	3532448
noclassa2	0	0	425	4697
parthi	0	6.5	0	790178
parthi1	0	2.3	0	279538
parthia2	0	0.3	0	43112
total	8.5	91.4	1035124	11096775

AA-quality for okay set (no okalt): all and longest 1000 summary

	n	average	median	min,max	sum	gaps
okay.top	1000	2096	1799	1372, 29738	2096967	344, 0.3
okay.all	638606	104	91	40, 29738	66481461	211449, 0.3
